# CyberLimb: a novel robotic prosthesis concept with shared and intuitive control

**DOI:** 10.1186/s12984-022-01016-4

**Published:** 2022-04-29

**Authors:** Nicolas Seppich, Nicholas Tacca, Kuo-Yi Chao, Milan Akim, Diego Hidalgo-Carvajal, Edmundo Pozo Fortunić, Alexander Tödtheide, Johannes Kühn, Sami Haddadin

**Affiliations:** 1 Chair of Robotics and Systems Intelligence, MIRMI-Munich Institute of Robotics and Machine Intelligence, Technical University of Munich (TUM), formerly MSRM, Munich, Germany; 2 Centre for Tactile Internet with Human-in-the-Loop (CeTI), Dresden, Germany

**Keywords:** Arm prosthesis, Assistive technology, Biomimetics, Cybathlon, Cybernetics, Human-centered

## Abstract

****Background**:**

Existing assistive technologies attempt to mimic biological functions through advanced mechatronic designs. In some occasions, the information processing demands for such systems require substantial information bandwidth and convoluted control strategies, which make it difficult for the end-user to operate. Instead, a practical and intuitive semi-automated system focused on accomplishing daily tasks may be more suitable for end-user adoption.

****Methods**:**

We developed an intelligent prosthesis for the Cybathlon Global Edition 2020. The device was designed in collaboration with the prosthesis user (pilot), addressing her needs for the competition and aiming for functionality. Our design consists of a soft robotic-based two finger gripper controlled by a force-sensing resistor (FSR) headband interface, automatic arm angle dependent wrist flexion and extension, and manual forearm supination and pronation for a shared control system. The gripper is incorporated with FSR sensors to relay haptic information to the pilot based on the output of a neural network model that estimates geometries and objects material.

****Results**:**

As a student team of the Munich Institute of Robotics and Machine Intelligence, we achieved 12th place overall in the Cybathlon competition in which we competed against state-of-the-art prosthetic devices. Our pilot successfully accomplished two challenging tasks in the competition. During training sessions, the pilot was able to accomplish the remaining competition tasks except for one. Based on observation and feedback from training sessions, we adapted our developments to fit the user’s preferences. Usability ratings indicated that the pilot perceived the prosthesis to not be fully ergonomic due to the size and weight of the system, but argued that the prosthesis was intuitive to control to perform the tasks from the Cybathlon competition.

****Conclusions**:**

The system provides an intuitive interface to conduct common daily tasks from the arm discipline of the Cybathlon competition. Based on the feedback from our pilot, future improvements include the prosthesis’ reduction in size and weight in order to enhance its mobility. Close collaboration with our pilot has allowed us to continue with the prosthesis development. Ultimately, we developed a simple-to-use solution, exemplifying a new paradigm for prosthesis design, to help assist arm amputees with daily activities.

## Background

Despite advances in electromyography (EMG) in the field of upper limb prostheses in recent years, myoelectric prostheses still cannot compensate for the loss of an upper limb entirely, due to, for instance, a lack of comfort or a non-intuitive control of the devices. As a result, an average of more than $20\%$ of users abandons their prostheses in daily life [1, 2]. High costs ($\$20,000-\$100,000$ on average) [3], which in many cases are required to be paid by the users themselves, makes the adoption of fully functional prosthetic devices unfeasible. However, designing an upper limb prosthesis that can be integrated into the daily life of a user with a high degree of comfort remains a complex challenge to research and industry.

Upper limbs provide a high number of degrees of freedom (DoF) and sensing capabilities to manipulate objects. They are essential for human dexterity. In case of loss, depending on the degree of amputation, finding a replacement yields high technological challenges to the mechanics and control of the prosthesis. The three most common degrees of amputation are transcarpal (amputation within the hand), transhumeral (amputation within the upper arm), and transradial (amputation within the forearm) in corresponding order [4].

Current research in myoelectric prosthetics focuses on control, as it is considered to be the biggest challenge by many researchers [3–5]. Mapping the signals of sEMG, a non-invasive technique measuring muscle activations [6], to kinematic movements with the help of pattern recognition promises in theory a natural sense of control. Well known state-of-the-art commercial prostheses such as the Michelangelo and Bebionic hand by Otto Bock [7], the i-limb by Össur [8] and the Hero Arm by Open Bionics [9] incorporate some form of this technology. Within the field, research has focused on feature engineering, e.g., signal amplitude and power, frequency information, or time series modeling and discriminative description [6, 10]. Additionally, deep learning-driven approaches that incorporate convolutional networks have gained traction in recent years. Rather than performing a manual handcrafting of features, the mapping between the signals and kinematic motion can be learned [11, 12].

Due to the fact that only limited information can be recorded with sEMG sensors at the same time through muscle activities, controlling more than one degree of freedom simultaneously may be physically exhausting for the pilot. Oftentimes, estimated gestures from muscle activation do not match the actual movement of a healthy arm. Additionally, long and strenuous training and calibration sessions are required for optimal performance [5], however the signal may deteriorate due to physiological factors such as sweating [4] or electrode displacement. Such factors might compromise the overall performance. Dexterity, and thus the ability to grasp, may also be inhibited by the complexity of the control required for the prosthesis. Furthermore, current commercial myoelectric prosthetic approaches do not necessarily reproduce the users’ intention. On the other hand, shared control uses machine intelligence to estimate the intention and reduce the control complexity on the pilot’s side. Previous researchers have shown promising results by combining additional visual information with semi-automatic control [13].

Under-actuated or soft adaptive grippers with one degree of actuation (DoA) address the issue of control complexity at the cost of reduced dexterity. Well-known anthropomorphic under-actuated grippers are the SoftHand Pro [14] with 18 DoF and 1 DoA, the Mia hand [15] and the Hannes hand [16]. Based on Festo’s Fin Ray®, non-anthropomorphic passive adaptive gripper designs with one DoA such as the work of [17] and [18], are promising concepts to conform to the shape of an object with high precision, which according to [4], is a limitation of many prostheses.

Challenges in prosthesis design also include aesthetic factors, trade-off between the weight (robustness and size of the battery) and sufficient grasping force (size of actuators), cost and access [3, 19]. Advances in 3D printing technology offer an alternative manufacturing technology that allows customized and especially less expensive designs, increasing the affinity for users [2] and allowing rapid prototyping and faster development.

In this work, we focused on reducing control complexity, finding a trade-off between weight, and grasping force, and reducing the cost of the final design, by developing CyberLimb, a trans-radial arm prosthetic system, which our pilot used to compete in the Cybathlon 2020 ARM Challenge. Cybathlon is a global competition for people with disabilities, in which they need to overcome tailored challenges resembling activities of daily living, by using body-powered or motor-powered prostheses in a constrained amount of time. The project CyberLimb was initially launched in April 2019 and the final version of the CyberLimb prosthesis resulted from a concept iteration that lasted less than 1.5 years. The final concept and prototype was developed and built in less than one year. CyberLimb was developed to provide the user with intuitive and semi-automatic control during the tasks of the Cybathlon competition. Overall, the CyberLimb system is intended to serve as an extension of the user’s arm to make daily tasks either possible or more comfortable to fulfill. At the same time, the system provides a research platform for engineers and pilots to develop enhanced solutions to help arm amputees perform everyday activities.

The main contributions of our work can be summarized as follows:

- Design and implementation of a low-cost 3D-printed 3 DoF prosthesis with a flexible, force-sensitive 1 DoF gripper, consisting of state-of-the-art electronics and robotic joints.
- A customized, intuitive and robust grasp control strategy selection through an FSR headband that recognizes muscle activation when the jaw is clenched.
- A machine learning algorithm for detecting different objects based on force signals from FSR sensors on the gripper.
- Shared-control features such as wrist flexion and extension auto-leveling, manual forearm supination and pronation and a smartphone-based user control interface.
- Validation and insights of the prosthesis prototype via training sessions and by participating in the 2020 Global Cybathlon competition.

## Methods

**Fig. 1:**
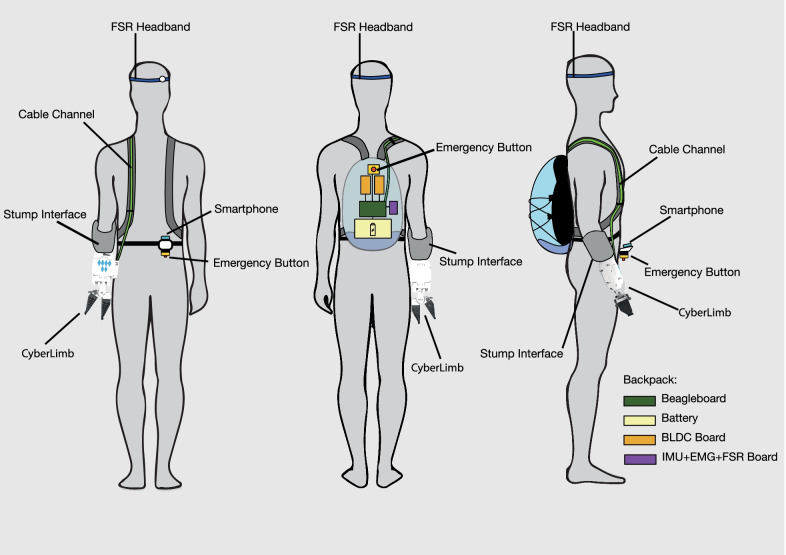
Overview: CyberLimb arm prosthesis system. The system consists of an arm prosthesis connected to a custom-made stump interface manufactured by OT Supply, a backpack containing the main controller, motor controllers and battery submodules, a smartphone-based control interface attached to the backpack waistband, and an FSR-based headband human-machine interface for gripper control

The CyberLimb system is depicted in Fig. 1 and consists of the following modules:

- An arm prosthesis connected to a specifically tailored stump interface manufactured by *OT Supply GmbH, Germany*.
- A backpack containing the computation unit based on the *BeagleBoard-X15* of the *BeagleBoard.org Foundation*, two developed brushless DC (BLDC) motor controllers, one sensor board, and one battery pack.
- A smartphone control interface with the phone *Samsung Galaxy S3* integrated onto the backpack waistband.
- An FSR-based headband as the human-machine interface gripper control solution.

The arm prosthesis with the stump interface weighs 1.95 kg in total with an overall length of 52 cm, a width of 12 cm, and a height of 9.5 cm. A comparison to the daily prosthesis is shown in Sect. 3. Through discussions and training sessions with our pilot, we developed a novel assistive device designed to provide an intuitive control interface to reduce physical and cognitive workload for our end-user. While developing the overall system, it was important to define goals early on in the design process to guide the device development.

### Goals

The main purpose of the CyberLimb system was to enable the pilot to accomplish as many tasks from Cybathlon competition as possible [20]. As the challenge tasks cover a variety of daily situations, a versatile system was needed to be developed. These tasks were subdivided into different gripping configurations, with different object geometries, and involving the sense of touch. The corresponding challenges were solved by designing a prosthesis with a suitable number of DoF, a versatile and flexible gripper geometry, and algorithms for smart movements and object detection with machine intelligence and task-dependent controls. The prosthesis also needed to be optimized for ergonomics and usability since a pilot would use it in the competition. Consequently, the weight, center of gravity, and limb interface were designed by localizing a majority of the weight proximally toward the stump interface to reduce the lever arm of the prosthesis on the pilot’s arm.

**Table 1:** Requirements used for the development of the CyberLimb prosthesis

Domain	Type	Requirement description
Mechanics	Kinematics	Available DoFs: 3 (Gripper grasp and release, Wrist flexion and extension and Forearm supination and pronation)
		Available parallel actuation of wrist and gripper
	Kinetics	Enough force of gripper and wrist to hold all objects of challenge
		Stable grasps, no slippage
	Geometry	Suitable geometry of gripper to grasp all objects of the competition with precision
		Available grasp types: cylindrical grasp and precision pinch
		Comfortable useful prosthesis with minimal weight and size
		Robust prosthesis design
		Prosthesis should be able to be built with limited resources: time, people and costs
Control	Functionality	Availability of a force regulated grasping option
		Availability of parallel control of gripper and wrist
		Availability of predefined prosthesis motions to complete tasks semi automatically whenever needed by the user
		Availability of haptic feedback
		Availability of orientation sensory information for shared control
		Grasping should be controllable by redundant inputs
	User experience	Possibility to switch fast between multiple control modes on user control interface
		Comfortable prosthesis to operate through an intuitive user control interface and non-tiring control of movements
		Easy to learn prosthesis operation
	Reliability	Control should show low latency and should be deterministic
		Prosthesis should automatically continue to hold an object once grasped to be failsafe

To solve the tasks of the Cybathlon Challenge 2020 and to derive requirements, a study was conducted in which a person solved each task with a healthy upper limb. The process of solving the task was then discretized into individual movements so that the technical requirements of the prosthesis could be abstracted and summarized quantitatively within a storybook. Table 1 gives a detailed overview of the resulting requirements based on the storybook analysis and our desired features for the prosthetic design.

Besides the technical developments, training of the pilot was also crucial for the competition. For the training, we replicated the competition set-up so that our pilot was able to train under the same conditions as in the Cybathlon competition [20].

A limitation described in the literature is the lack of direct proportional sensory feedback that gives the user a sense of touch. This was a crucial requirement for solving the Haptic Box task from the Cybathlon competition, where different shapes and materials of objects should be felt without visual contact. To fulfill this task as robustly as possible, we decided not to encode the information into direct proportional feedback that would be transmitted to the pilot directly. Instead, we decided to transmit only the interpretation of the shape and the hardness of the objects. Section 3 explains the details.

A crucial insight we observed during our preparations for the competition was that we needed to tailor our solutions to both our pilot and the competition itself. In our final design, we opted not to use sEMG-based control. The weight of our device (1.95 kg) made using this control strategy difficult for our pilot, who was used to a lighter (1.03 kg) prosthesis. However, through discussions with the pilot, we determined that a head interface would be a simpler and intuitive solution. She ultimately preferred our FSR headband solution because it required less physical and cognitive effort to operate.

### Prosthesis design and materials

**Fig. 2:**
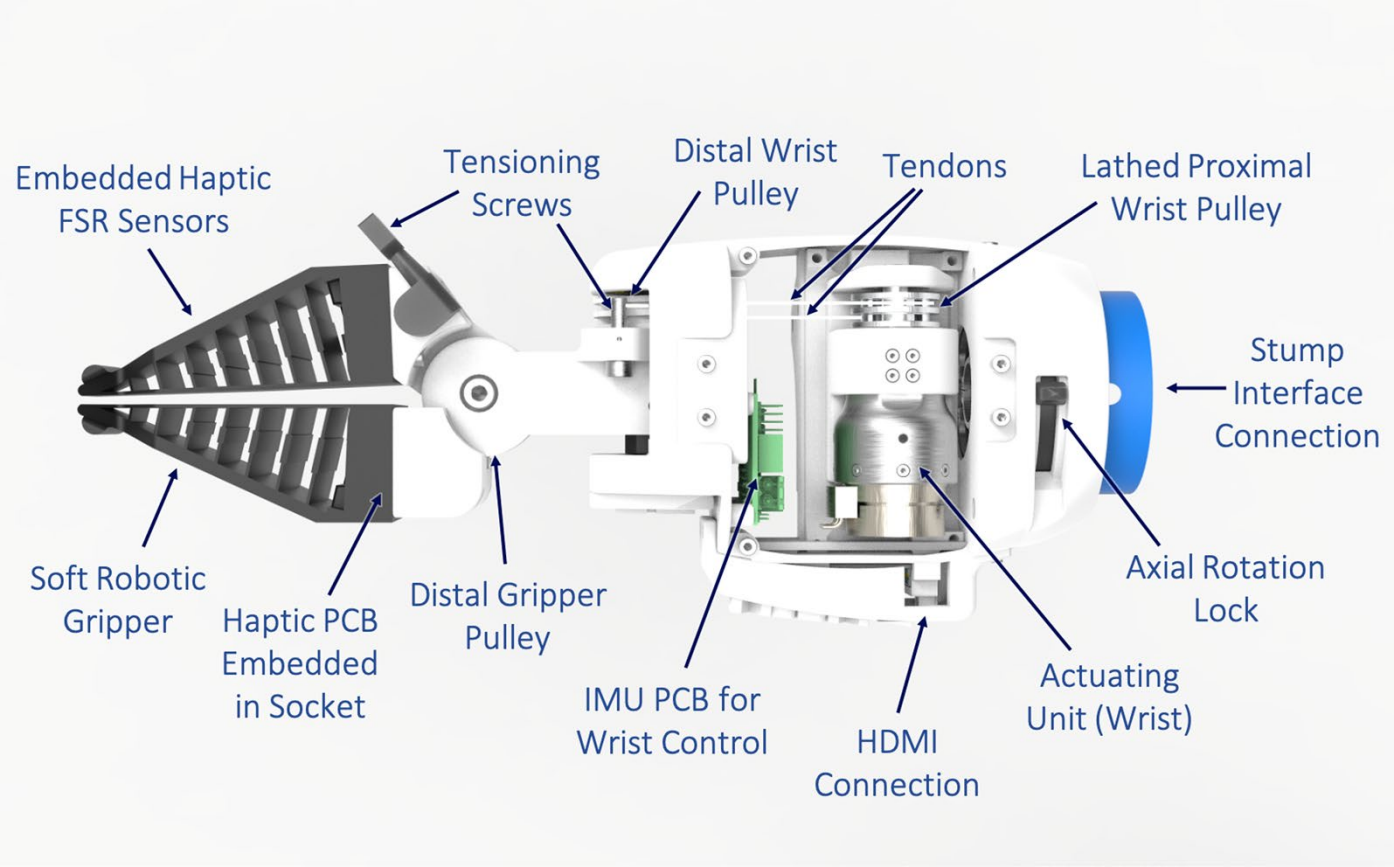
Top view of the arm prosthesis (CyberLimb) with top and bottom lids removed. The wrist actuating unit is visible, which drives the proximal wrist pulley attached to the distal wrist pulley with tendons to actuate the wrist-based on positional measurements determined from the inertial measurement unit (IMU) sensor housed in the prosthesis. The wrist can flex and extend ±50$^{\circ }$ and is able to maintain a horizontal gripper position in the horizontal “auto-leveling” mode. An identical actuating unit is positioned directly below the wrist actuating unit shown. This actuating unit drives the proximal gripper pulley attached to the distal gripper pulley with tendons to open and close the gripper based on user control from the FSR headband or phone control interface. Tensioning screws are fixed to the gripper and wrist for quick access to tendon tensioning. The grippers are 3D printed from a flexible thermoplastic polyurethane (TPU) material allowing them to conform to different object shapes. FSR sensors are embedded directly into the gripper body in order to differentiate objects and thus provide feedback to the user. A sliding lock allows the user to lock the axial forearm rotation position in place. When unlocked, the forearm can supinate and pronate relative to the stump interface to change the rotational position of the gripper. An HDMI connection port is fixed to the side of the prosthesis to connect to the electronics housed in a separate backpack

Based on task-oriented approaches from the Cybathlon competition, we developed a prosthesis designed to handle complex tasks while providing an intuitive user interface. Figure 2 shows a top view of the arm prosthesis with the top and bottom lids removed. Rather than directly mimicking the biological hand requiring a large bandwidth of control data, we focused on a solution utilizing a simple soft-gripper that is able to adapt to a wide range of tasks and objects. An automatic wrist flexion and extension control was incorporated into the design to enable the user to manipulate the device in varying positions without compromising the gripper position. In cases where the horizontal gripper was not suitable, the forearm could be manually supinated and pronated about the stump interface to axially adjust the gripper. With this simple gripper-based approach, the prosthesis design was able to handle all Cybathlon tasks with minimal need for adjustment from the nominal horizontal gripper position.

**Fig. 3:**
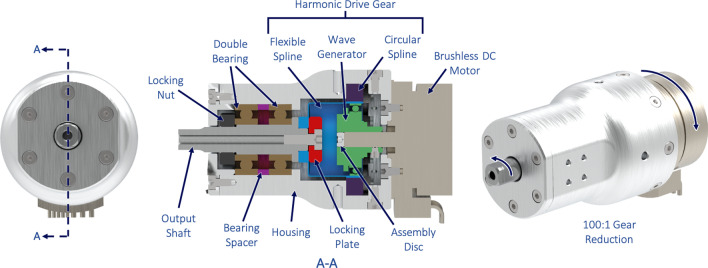
Custom-made actuating unit for high fidelity control with optimized size and weight ( ø2 7 $\times$ 59 mm). The actuating unit consists of an 100:1 harmonic drive (HD) gearbox driven by a BLDC motor, which combined can produce an output torque of approximately 4 Nm. The resulting output shaft rotation is opposite to that of the input motor rotation

To provide high torque and high fidelity low-level control, custom actuating units were developed for backlash-free movement of the prosthesis (Fig. 3). Each unit consist of a brushless DC motor (Faulhaber) with embedded hall sensors and a harmonic drive (HD) gearbox. Housing components and output shaft were manufactured from aluminum, providing a lightweight but rigid interface. Two actuating units are used in the device. They are located at the forearm and control the wrist flexion and extension movements, as well as opening and closing of the gripper, respectively (see Fig. 2). Both actuating units are stacked and placed as close as possible to the stump interface to reduce the lever torque from the prosthesis. The motor within the actuating units operates at a rated torque of 41 mNm, rated speed of 6200 rpm, and a rated voltage of 24 V. The HD gears have a gear ratio of 100:1 and an output torque of about 4 Nm. Despite the high cost, making them the most expensive components of the prosthesis, the HD gears offer advantages over the alternative power transmission gear types e.g. cycloidal or planetary gear units. As such, an HD represents the state-of-the-art and can deliver gear reduction ratios from 30:1 up to 800:1 [21]. Due to their large gear meshing area, HDs have a torque capacity comparable to conventional drive solutions of twice as much the volume size and three times as much its mass [22]. Therefore, HDs are often favored for electro-mechanical systems with space and weight constraints [23].

Particularly in our use case the HD’s lower weight also shows benefits in terms of user experience.

Cycloidal gearboxes, on the other hand, have a similar reduction ratio with comparable geometric compactness, but due to their eccentric nature, they tend to generate vibrations, which ought to be absorbed by the pilot’s arm stump and is therefore an unfeasible solution. In summary, we favored the pilot’s convenience and thus equipped our prosthesis with an HD opting for its unique performance features, such as high gear ratios, high torque capacities, zero-backlash, and compact geometry [24].

**Fig. 4:**
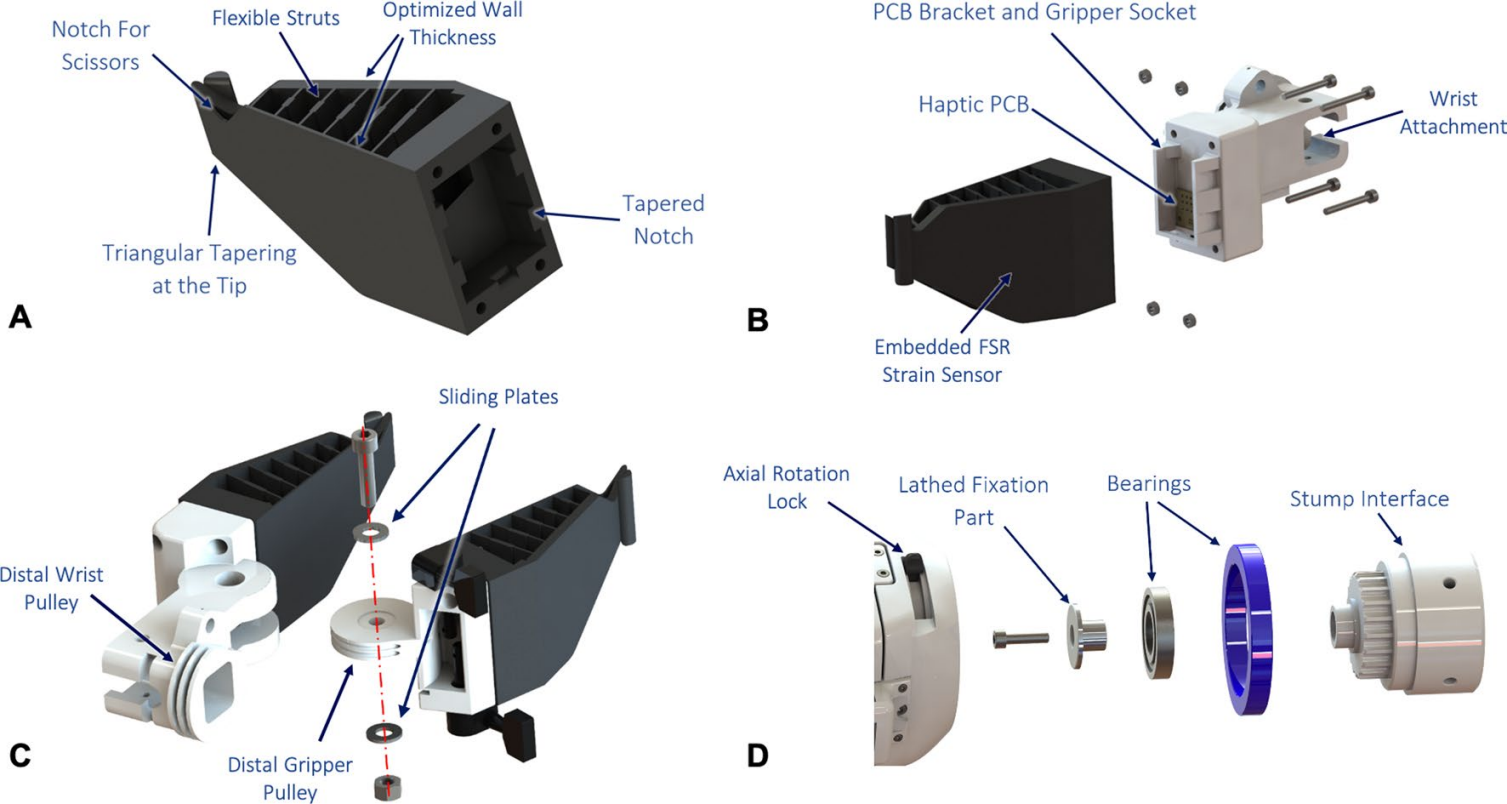
CyberLimb arm prosthesis device mechanics. **A** Soft robotic gripper 3D printed from flexible TPU material. Flexible struts are incorporated into the gripper to allow it to flex around various sized objects. A notch is cut at the tip of the gripper to be able to grasp the scissors from the competition task. The gripper has a tapered shape to have adequate surface area for gripping objects. **B** FSR sensors are embedded into the gripper body to be able to differentiate between disparate types of objects based on measured contact forces. The gripper attaches to the wrist shaft, which houses the printed circuit board (PCB) that reads FSR sensor measurements. **C** Close up of exploded gripper joint view depicting distal gripper and wrist pulleys. The tendons are routed through the prosthesis forearm to drive gripper and wrist actuation, respectively. **D** Exploded view of the axial rotation mechanism. A sliding lock allows the user to lock axial forearm supination and pronation in place with respect to the stump interface. Notches on the stump interface connector allow incremental rotation of the prosthesis’ forearm body. The mechanism is fixed with a lathed aluminum cap and a double bearing rotation joint

**Fig. 5:**
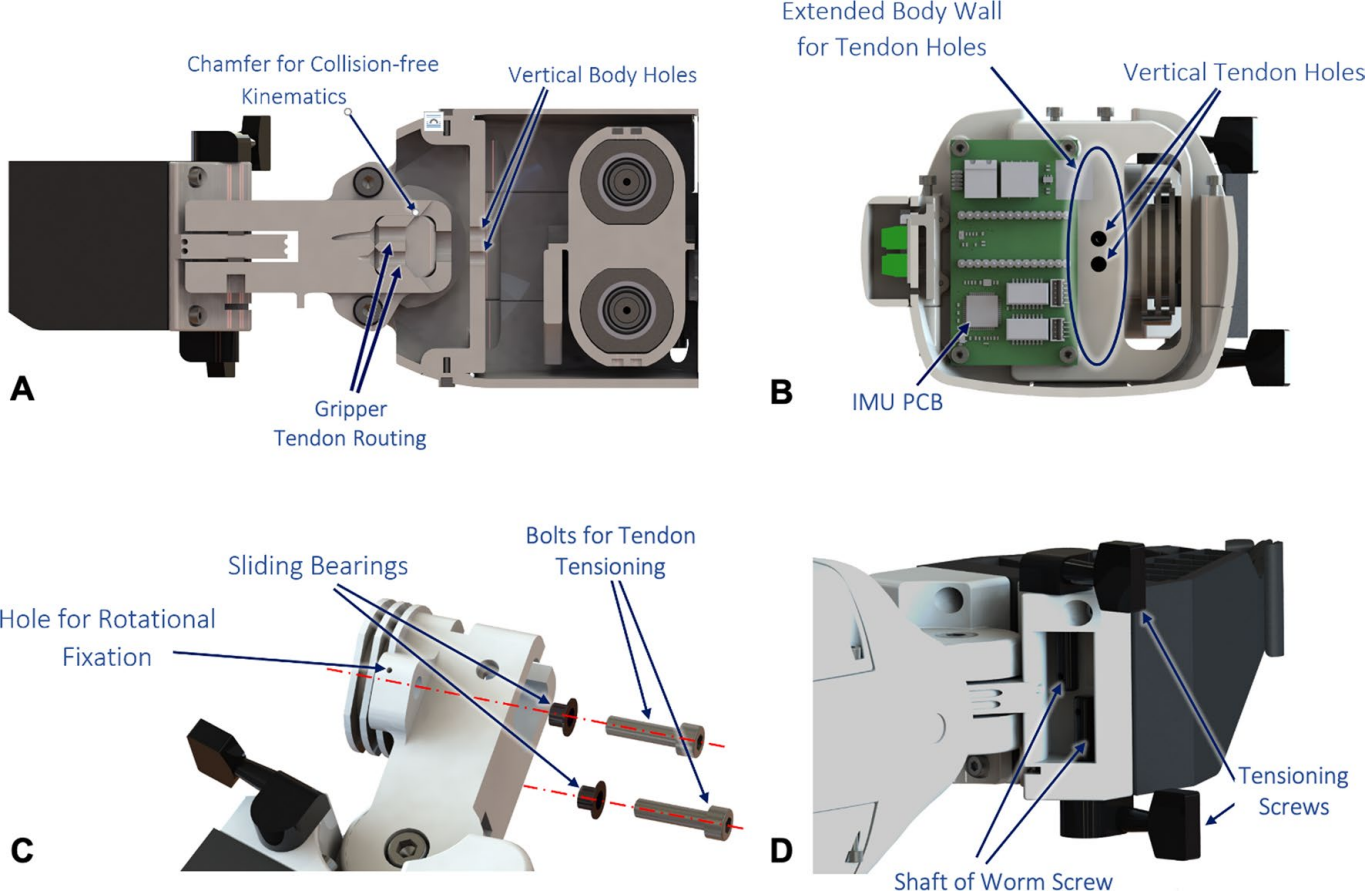
Tendon routing and tensioning. **A** Side section view of the prosthesis showing two vertical holes for gripper tendons. The tendons continue through the central wrist shaft and wrist connection that has a large chamfer to prevent collisions with the tendons at extreme wrist angles. The tendons continue through the wrist joint to wrap around the distal gripper pulley and are fixed to the tensioning screw. **B** Axial section view showing the two vertical holes directly next to the IMU PCB through which the gripper tendons pass through. The wrist tendons go directly through the large hole in the body wall and wrap around the distal wrist pulley shown. **C** The wrist tendons are wrapped around the pulley and fixed with bolts adjacent to the pulley. These bolts are used for tensioning the wrist tendons. **D** Close-up view of the tensioning screws that tighten the gripper tendons. The gripper tendons wrap around the gripper pulley and feed into the shaft of the worm screw. As the guitar knob is rotated, it drives the worm screw to tighten the gripper tendons

The gripper consists of a two-finger soft robotic design (Fig. 4A–C). The gripper fingers are 3D printed with a flexible TPU material (NinjaFlex 85A) and perpendicular struts that allow it to conform as it grasps objects. This provides a maximum contact surface for enhanced gripping regardless of the shape of the object to be grasped. Additionally, a rubber padding was mounted to the inside face of the gripper to achieve robust grasping with high friction and low grasping force. With this setting, a maximum normal force of 63 N can be applied to hold objects, which is equivalent to the force of the Mia hand (70 N) [15] and higher than that of the Bebionics hand (36 N) [25]. FSR sensors (with a sensing range up to 100 N) are embedded within the outside walls of the gripper to be able to identify the shape and material hardness of the grasped objects based on measured contact forces. To open and close the gripper, a tendon-based actuation mechanism was implemented (Fig. 5A, B). Tendons fixed on the proximal gripper pulley are fed through two vertical holes in the forearm body. Next, the tendons continue into the wrist shaft and through a chambered opening in the wrist attachment until they are finally wrapped around the distal gripper pulley and fixed to the tensioning screw (Fig. 5D). As the actuating unit in the forearm drives the proximal gripper pulley, the tendon pulls, thereby rotating the distal gripper pulley to close the gripper. Conversely, when the actuating unit reverses direction, the opposite tendon pulls to open the gripper. The tensioning screw consists of a tuning knob from a guitar and is mounted to the gripper attachment. When needed, the tensioning screw can be tightened to subsequently tension the tendons as needed via a worm screw mechanism.

Additionally, a tendon-based actuation mechanism was implemented for wrist flexion and extension. The wrist actuating unit drives the proximal wrist pulley directly connected to the distal wrist pulley via tendons. A large gap in the forearm body wall allows the tendons to be fed directly between proximal and distal pulleys (Fig. 5B). The tendons wrap around the distal pulley are subsequently fixed to external bolts used for tensioning (Fig. 5C). As the wrist actuating unit drives the proximal pulley, the tendons pull causing the distal wrist pulley to rotate, thereby actuating the wrist-gripper sub-assembly for flexion and extension. This allows the wrist to flex and extend between ±50$^{\circ }$. Similar to the gripper tendon mechanism, the tendons are able to be tensioned as needed by tightening the external tensioning bolts.

An axial rotation mechanism at the forearm-stump interface connection was developed (Fig. 4D). This allows the user to manually supinate and pronate the prosthesis body. Grooves were incorporated into the stump interface connector to allow the user to incrementally adjust the rotation angle. Additionally, there is a sliding lock on the side of the device, which allows the pilot to lock the prosthesis rotation in place at a given angle. When unlocked, the device is able to be manually supinated or pronated with the other arm to adjust the gripper orientation. The mechanism is held together with a lathed aluminum cap and double bearing joint to provide smooth rotation.

The stump interface was manufactured and provided by *OT Supply* who specializes in building equipment and prostheses. The company has previously manufactured the stump interface for the prosthesis that our pilot uses in her daily life. A silicone liner is located between the interface and the arm to ensure that the prosthetic arm does not slide out, as well as to provide a soft interface between the pilot’s arm and the prosthesis. This stump interface was fixed to the end of the rotation mechanism.

The embedded electronic subsystem of the prosthesis is comprised of the sensor acquisition submodule and the actuation control submodules. The Sensor acquisition submodule process the signals from the FSR sensors located in the soft gripper fingers and headband, the IMU sensor located on-board and two sEMG channels located on the stump interface (not used in the end). The main PCB is located inside the forearm (Figs. 2, 5B) and two auxiliar PCBs for easy wiring are located within the connection between the soft gripper and the gripper attachment (Fig. 4B). Two actuation control submodules for the control of the motors are located in an additional electronics box housed in a small backpack (Fig. 1) that the pilot wears to prevent additional weight within the prosthesis body (Fig. 1). An HDMI connection to this box is located directly on the prosthesis at the forearm side (Fig. 2). This connection rotates together with the prosthesis relative to the stump interface. This ensures that the rotation does not have any effect on the wires connected to the PCBs to avoid any wire tangling.

**Fig. 6:**
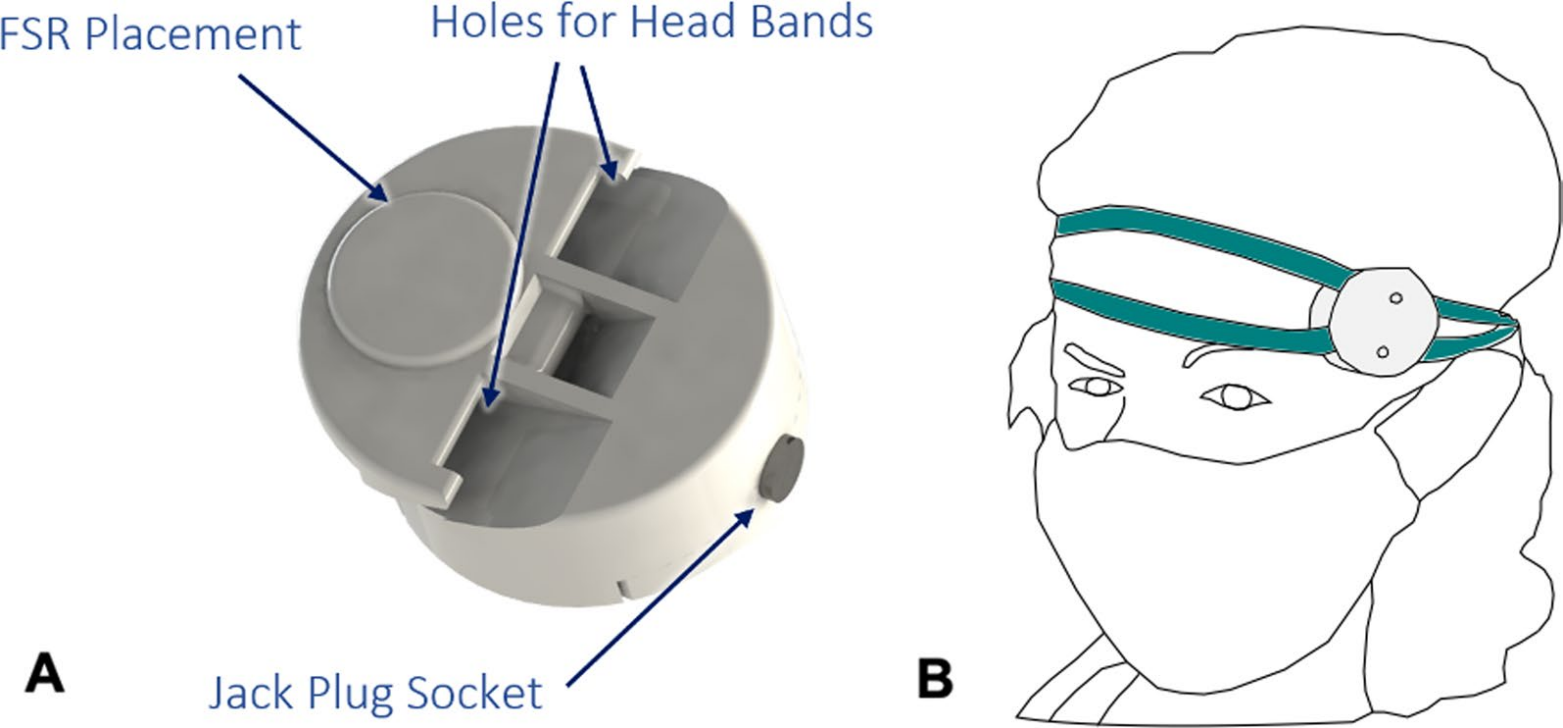
FSR Headband **A** Close-up view of the FSR headband attachment. The FSR sensor is placed on the edge of the 3D printed attachment. Two holes on the attachment allow a headband to pass through for mounting the device on the head. **B** Schematic of the FSR headband mounted to the pilot’s head. The FSR sensor is positioned on the temple of the pilot so that when she clenches her jaw, a force is applied to the FSR sensor. When a certain force threshold is exceeded, a binary signal is extracted to trigger an action of the gripper—either closing or opening depending on the gripper’s state

The prosthesis also contains a headband with an FSR sensor used to control the gripper (Fig. 6) and a smartphone (Samsung Galaxy S3) fixed to the backpack strap for additional control options and for adjusting the prosthesis settings (Fig. 1). Details of the control system pertaining to the architecture and user interface via the FSR headband and smartphone application are detailed in the next section. Lastly, an external box containing the battery unit is also stored in the backpack.

### Software and control architecture

**Fig. 7:**
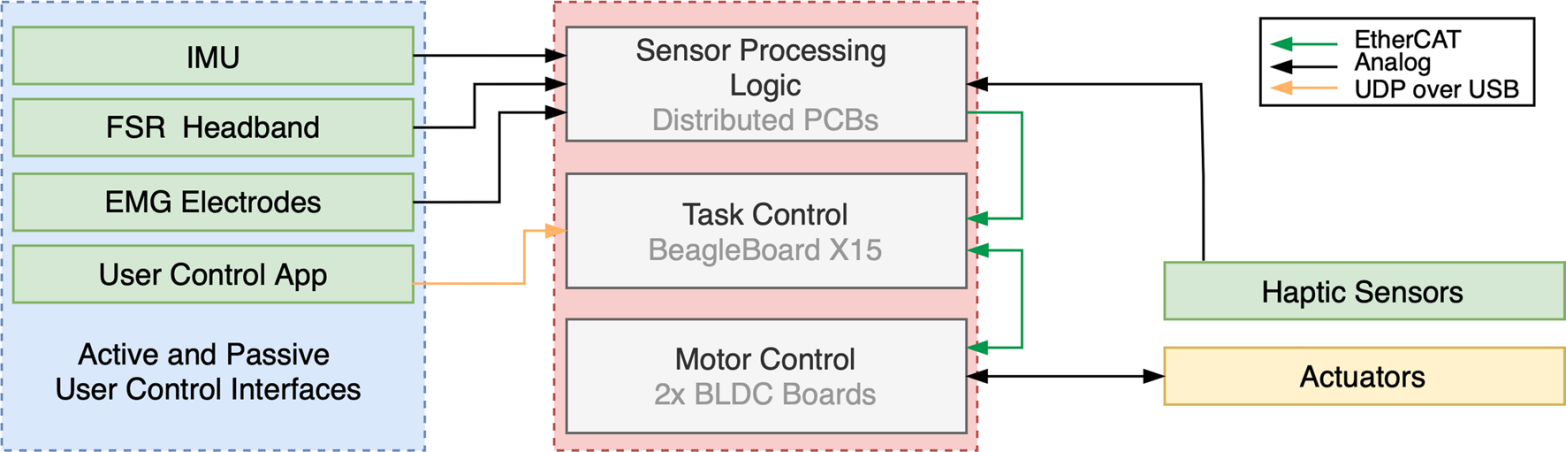
Block diagram of the system control and communication architecture

Figure 7 depicts the overall software and communication architecture. The high-level task control logic software is implemented using MATLAB Simulink and runs on a BeagleBoard-X15 [26], with a TI Sitara AM 5728 SoC based on a dual-core ARM Cortex-A15 processor that runs at 1.5 GHz. The main control loop runs at 1 kHz and connects to the electronic submodules via EtherCAT in real-time. Additionally, it connects directly to the User Control App running on a Smartphone via USB through UDP messages. Each Actuation Control submodule handles the control of each BLDC motor. The low-level cascaded control of the motor runs at 12 kHz and is implemented via an inner current control loop and a outer position control loop using the commonly used six-step-commutation technique.

**Fig. 8:**
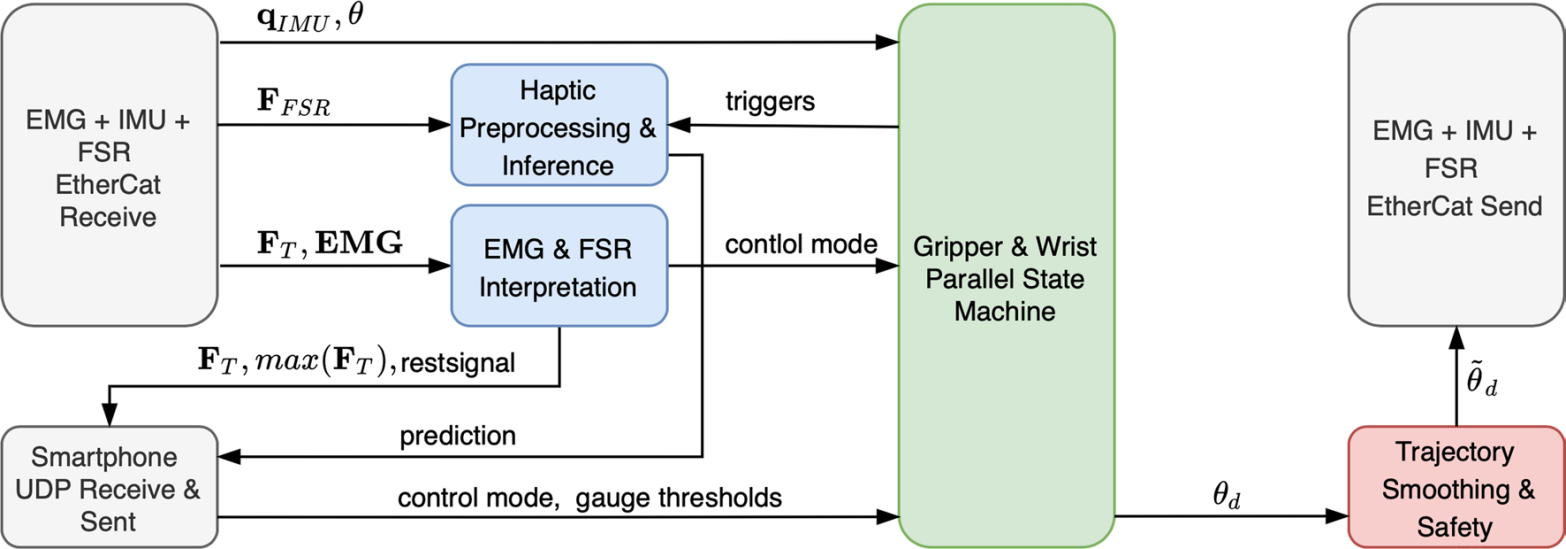
Block diagram of the task control architecture and signal flow implemented in Simulink

**Fig. 9:**
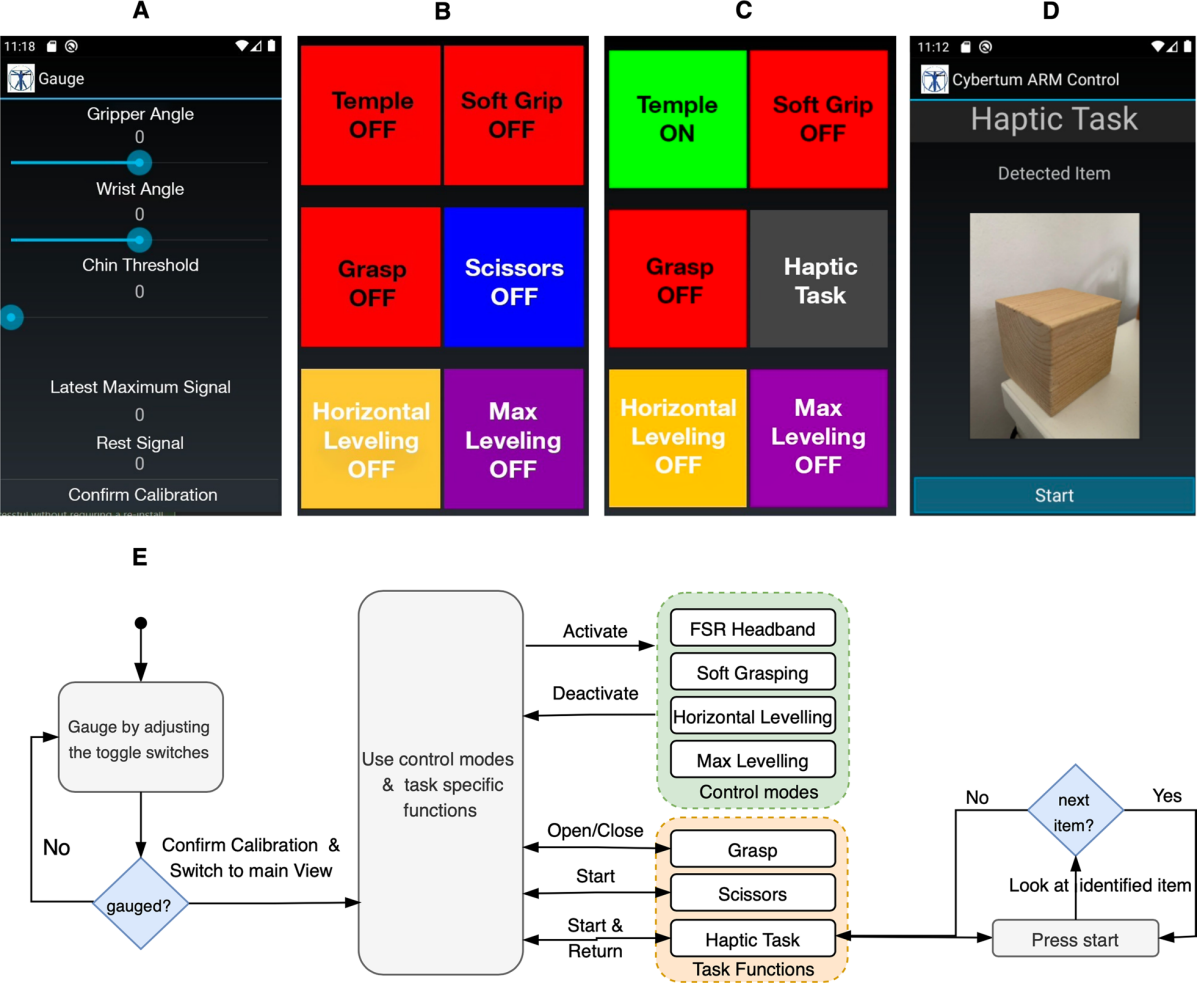
User Control App and workflow. **A** Gauge page to set up the calibration of the actuators and the FSR headband threshold—depicted as “Chin Threshold”. **B** Main view of the user controls switched off. **C** FSR headband control is switched on and changed to Haptic View by holding down the scissors button. **D** Haptic page depicts a detected item after pressing the haptic button and starting the haptic algorithm. **E** Overall workflow of user Control App depicting the initial gauge calibration, main usage application, and haptic task function

High-level task control translates user inputs into commanded joint angles based on the state of the prosthesis. The core unit of task control is a finite state machine handling the desired angles of the gripper and the wrist in parallel (Fig. 8). The desired angles are calculated based on current joint angles $\mathbf {\theta } \in {\mathbb {R}}^2$, shaft orientation ${\mathbf {q}}_{IMU} \in {\mathbb {C}}^4$ and the control mode (either via smartphone interface or via interpreted $\mathbf {EMG} \in {\mathbb {R}}^2$ signals). A trajectory smoothing and safety unit avoids signal jumps and ensures a maximal rate of change in the output signal $\mathbf {\tilde{q}_d}$.

The primary commands that control the finite state machine are sent from the smartphone app—the central user interface (UI). Figure 9E depicts the workflow of the Smartphone UI. To enable the usage of the prosthesis, it first needs to be calibrated. Initial thresholds for the gripper and wrist are set in order to place the prosthesis in its initial configuration (gripper closed & wrist straight) via the calibration view of the Smartphone UI (Fig. 9A). Additionally, the FSR headband threshold is set either automatically or manually based on the received reference signal of the user at rest and the average value of the maximum read force when the pilot tenses her jaw muscle. Subsequently the user selects the control mode in Control View (Fig. 9B).

The UI contains general control modes and task-specific functions to use the prosthesis based on user preference. General control mode provide functionality that can be activated or deactivated. To choose the FSR headband mode, which is the primary UI for grasping control, the button “Temple OFF” needs to be pressed. In this mode, the grasping and opening of the gripper is toggled by exceeding the calibrated threshold for the force measured by the FSR sensor ${\mathbf {F}}_T$, “Soft Grasp” also activates the FSR sensors ${\mathbf {F}}_{FSR}$ in the gripper to ensure that a certain contact force is not exceeded. The wrist may be passively controlled by the pilot through an automatic control scheme or adjusted to be rigid. Based on the measurements of ${\mathbf {q}}_{IMU}$, the control scheme can decode the user’s intentions for automatic wrist adjustment. In “Horizontal Leveling” mode, the wrist will automatically adjust to keep the wrist horizontally leveled at all times to support object manipulation. In “Max Leveling” mode, the wrist is in maximum extension and rotates to maximum flexion if the prosthesis roll angle defined through ${\mathbf {q}}_{IMU}$ exceeds $\pm 90^{\circ }$. This supports the pilot especially in the cup stacking task of the Cybathlon to help flip the cups.

As a redundant option to grasping via the FSR headband, the pilot can also open and close the gripper using the phone interface by pressing “Grasp”. Additional task-specific functions such as “Scissors” or “Haptic Task” trigger a sequence of automatic wrist and gripper movements to solve the corresponding task. The buttons for “Scissors” and “Haptic Task” can be toggled by a long press on the respective button.

### Haptic feedback system

To solve the Haptic Box task of the Cybathlon competition, a sensory feedback system was required. The challenge consists of identifying a sequence of 5 objects $o_i$ out of 6 object classes of specific shape, texture, and compliance without visual feedback. Our approach entailed measuring contact forces and bending of the flexible gripper during one grasping cycle with 8 FSR and 4 bending sensors in addition to the current gripper configuration $\theta _1$. Figure 4B depicts the integration of the sensors into the gripper. The resulting individual object fingerprint is classified with a machine learning algorithm. The result is shown to the user via the smartphone application. The Haptic Preprocessing and Inference Module, depicted in Fig. 9D, E, implements this functionality. The underlying procedure is explained in the subsequent algorithm 1.

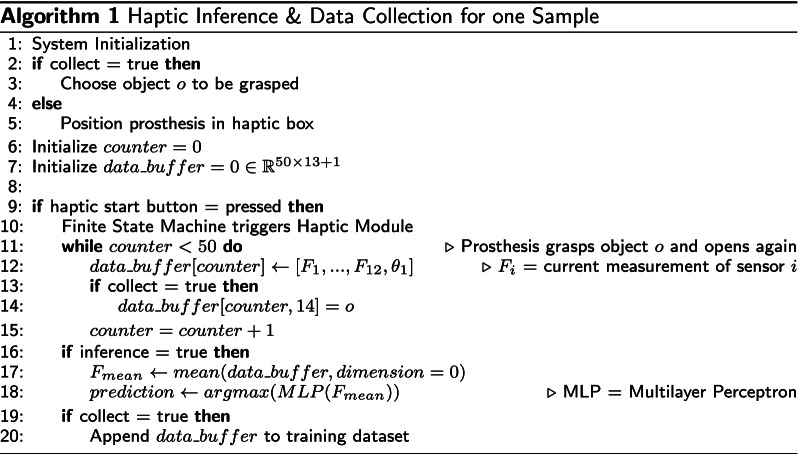



**Fig. 10:**
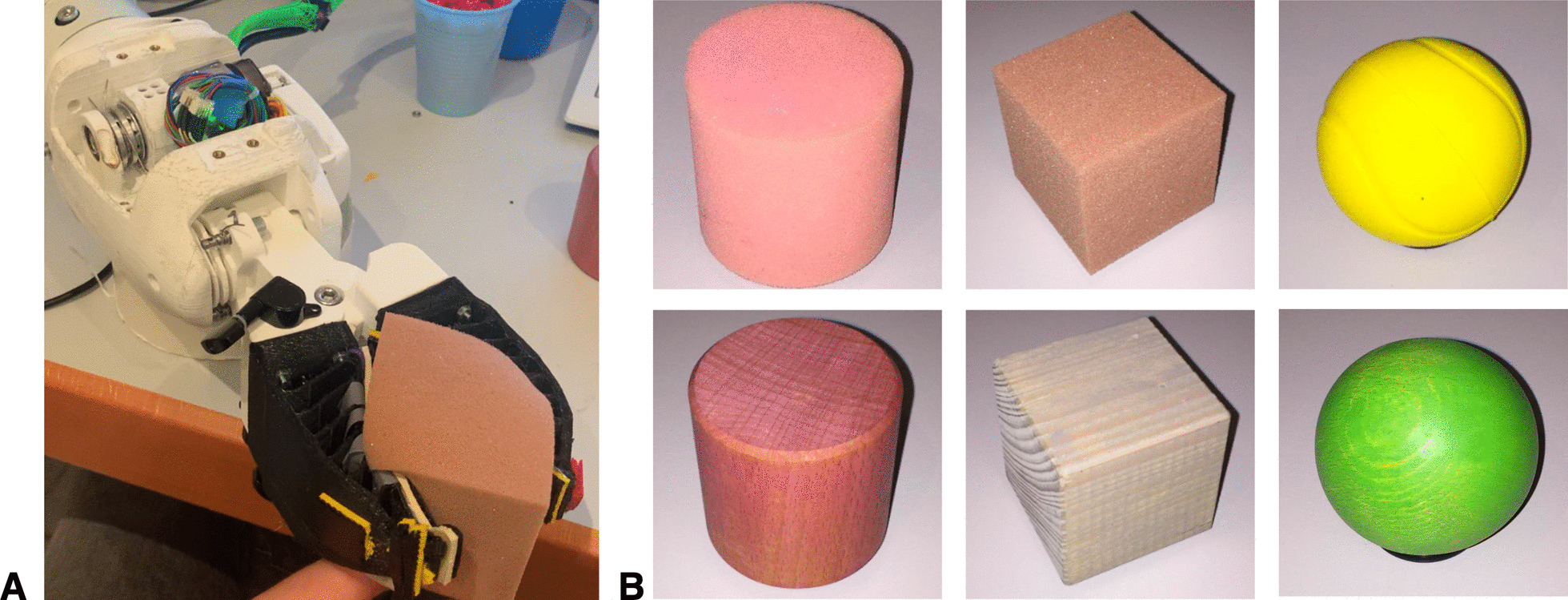
Haptic task training example. **A** Recording a haptic data sample for a soft cube. **B** Objects from the “Haptic Box” task to be classified without visual feedback

**Fig. 11:**
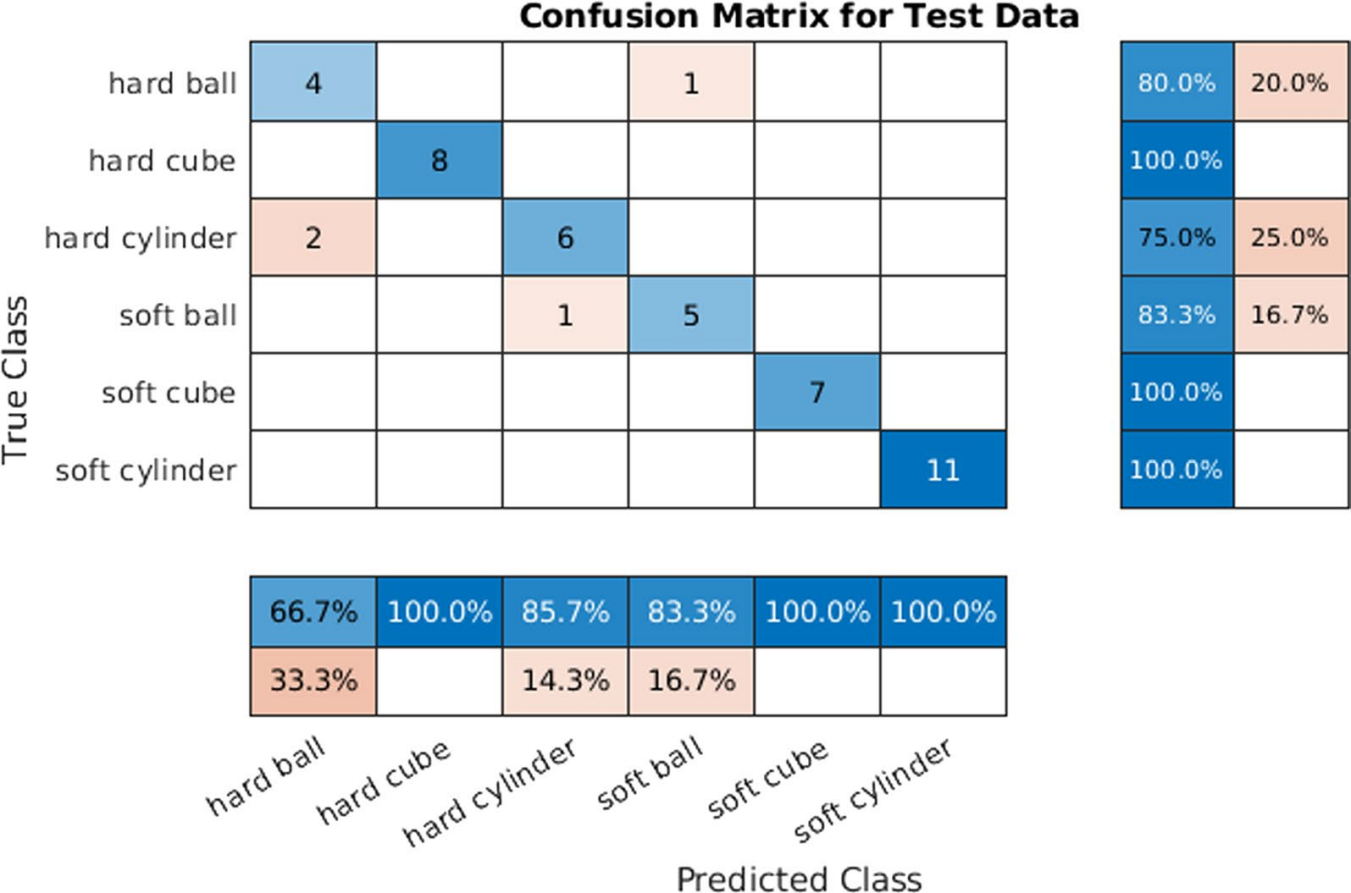
Confusion matrix of the test dataset. It determines the performance in classifying objects of the haptic box tasks

To collect the dataset to train our algorithm, 300 grasping cycles were performed, where the prosthesis had to grasp the 6 objects in a uniform frequency with random orientation (Fig. 10). The resulting dataset was split into train, validation, and test set with a respective ratio of 70, 15, and 15 percent of the data. Due to its computational efficiency and sufficient performance, a multilayer perceptron (MLP) was selected. The MLP was trained using the scaled conjugate gradient back-propagation algorithm [27]—an algorithm integrated in MATLAB. The hyperparameter search on train and validation set with 300 samples resulted in the architecture of: 4 layers with 50, 150, 100 and 40 neurons respectively. An L2 regularization of 0.05 was used. With this, a train accuracy of 100%, a validation accuracy of 91.1 % and a test accuracy of 91.1% could be achieved. The test accuracy could be further specified for individual objects. From Fig. 11 we can conclude that the network was able to distinguish between hard and soft objects with two outliers. It classified the objects hard cube, soft cube and soft cylinder without error. The round objects were more difficult, e.g., the hard cylinder was wrongly classified as a hard ball. Besides this high accuracy, we acknowledge that the dataset is rather small due to the complexity of the recording process and point out the low robustness to sensor failures leading to deviating results.

### Evaluation of prosthesis performance

A single prosthesis user with right-arm trans-radial arm amputation (>1 year) evaluated the CyberLimb prosthesis in training sessions and by participating with the device in the Cybathlon Global competition 2020. Qualitative findings based on feedback from the pilot in addition to task success rate were recorded while attempting to complete tasks from the Cybathlon competition (Breakfast, Clean Sweep, Home Improvement, Haptic Box, and Stacking Cups tasks) [20]. Following participation in the competition, a usability questionnaire was given to the pilot to evaluate usability factors pertaining to the CyberLimb prosthesis compared to her daily use prosthesis.

## Results

### Findings from training and competition

The training initially began with the pilot controlling the prosthesis’ gripper using two EMG sensors placed on the forearm. However, due to a limited training time (22 hours) combined with the excessive weight of our prosthesis and the difficulty of the race track, the pilot did not adapt well to EMG control and claimed it to be fatiguing in the long-term. To address this drawback, we used an alternative human machine interface for controlling the gripper, namely the FSR headband. Since the temporal muscles can be controlled by mastication, this solution did not impose additional strain on the arm.

**Fig. 12:**
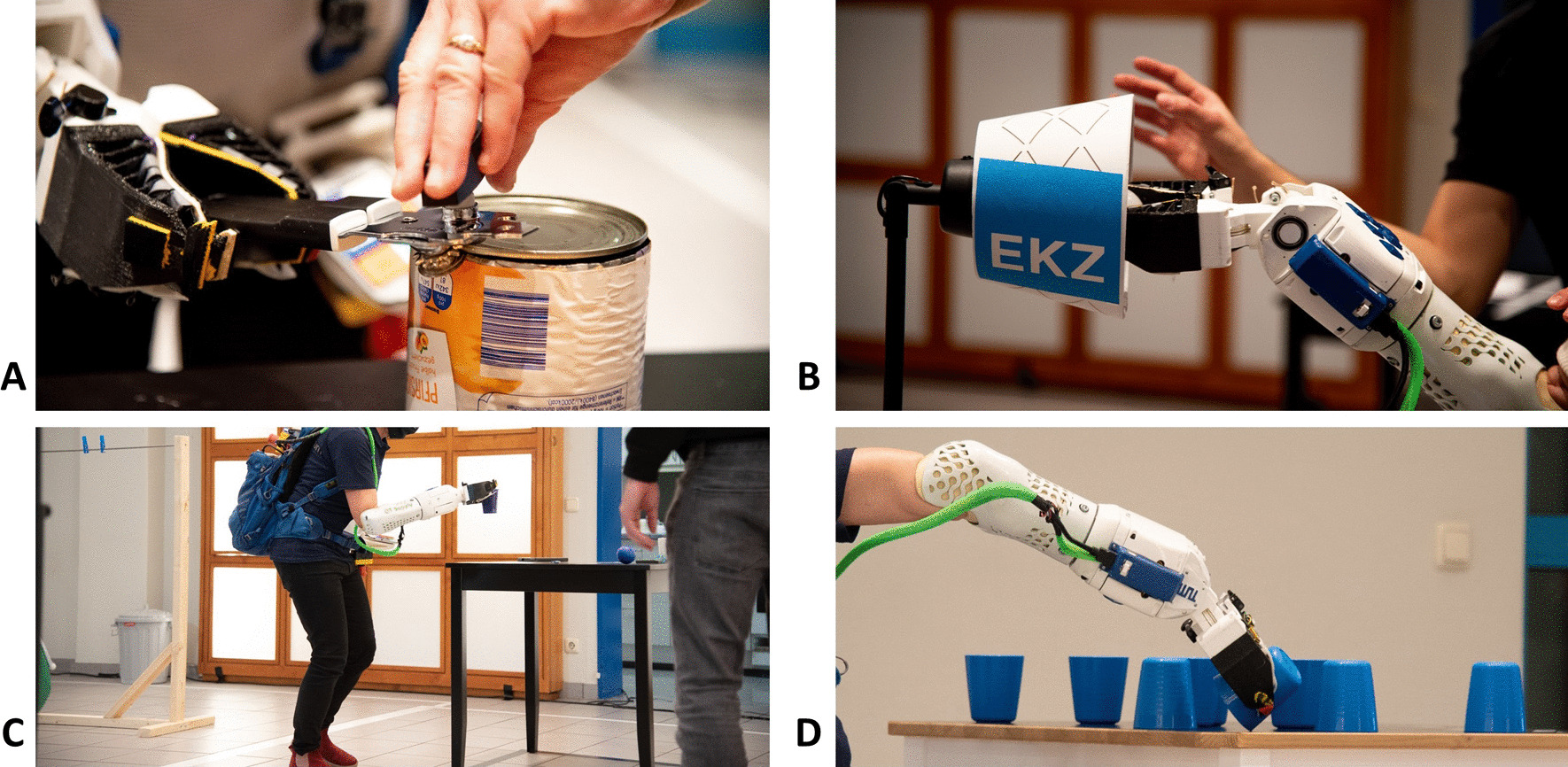
Pilot performing tasks from the Cybathlon competition and training sessions. **A** Opening a tin can using the can opener in the “Breakfast” task. **B** Screwing in the lightbulb using the wrist auto-leveling mode in the “Home Improvement” task. **C** Using the soft grip mode for carrying the plastic cup with small balls in the “Clean Sweep” task. **D** Flipping cups using an automatic wrist adjustment in the “Stacking” task

During training sessions the system needed to be updated based on user experience for solving the tasks more efficiently. Within the breakfast task, the initial assumption was to use the gripper to grasp the lever of the can opener while the other hand squeezed its handles. However, this strategy was uncomfortable and demanding for the pilot. As a result, we practiced switching hands, where the prosthesis squeezed the handles, and the other hand turned the lever (Fig. 12A).

For the “Clean Sweep” challenge, the horizontal auto-leveling of the wrist flexion and extension was especially useful to help keep the carried items horizontally. Additionally, when grasping the plastic cup filled with balls, the pilot set the device to the soft-grip mode to grasp the cups with a lighter grip hland avoid over squeezing (Fig. 12C). This task required physical fitness and concentration as the pilot needed to hold the items securely in the gripper while running between two tables. The soft-grip mode was suitable for the challenge; however, for daily usage, the grasping force would need to be adaptive to the situation.

Another challenging task for the pilot was screwing in a lightbulb as part of the “Home Improvement” challenge. Reaching above her head and positioning the lightbulb carefully in place was difficult for her due to her height. As a result, we practiced using the horizontal auto-leveling mode, which ensured proper horizontal positioning of the lightbulb into the lamp (Fig. 12B). The flexed wrist allowed her to screw in the light bulb from a comfortable position, instead of raising her entire arm above her body while rotating the prosthesis. In combination with the horizontal auto-leveling mode, after the initial start, the pilot could fix the wrist in the horizontal position and then manually supinate the prosthesis axially to screw in the lightbulb. The pilot was required to do multiple grasps and releases of the lightbulb to screw it in entirely.

Additionally, while at first having difficulties, the pilot was able to hammer in the nail during training sessions. This particular task led to the incorporation of the rubber pads on the gripper for a large friction surface area to handle the high impact on the prosthesis. For the overall “Home Improvement” challenge, the most difficult subtask was using the scissors. With the current gripper geometry, it was mechanically difficult to grab the scissors. We implemented a notch (Fig. 4A) to address this issue. Moreover, a task-specific control mode supported the pilot to grab the scissors and execute the paper cutting. During the competition, the pilot attempted the “Home Improvement” challenge, but was not able to finish all tasks successfully.

In the “Stacking Cups” task, we originally hypothesized that the pilot would be able to rotate her body with the prosthesis to flip the cups. However, after observing training sessions and receiving user feedback, we decided to change our strategy in order to reduce the energy expenditure and retain comfort for the pilot. Having addressed this problem, we implemented a shared-control option for this task. The Max Leveling mode was used to set the wrist angle from a fully flexed position to a fully extended position depending on the prosthesis arm angle relative to the ground. This shared-control method proved to be much simpler for the pilot to perform the cup flipping. Figure 12D depicts the pilot flipping the cups using the Max Leveling mode. From this position, the wrist automatically adjusts to full extension, thereby flipping the cup for her.

Overall, the pilot was able to accomplish the “Clean Sweep” task (Fig. 12C) and the “Stacking Cups” task (Fig. 12D) in the competition. While she was able to accomplish the “Breakfast”, “Home Improvement”, and “Haptic Box” tasks during training sessions, she was not able to accomplish these during the competition due to time constraints and due to a sensor failure of the haptic sensors. The “Laundry” task was never attempted due to the inability to put on the sweatshirt while wearing a backpack. Despite some of these limitations, the pilot demonstrated ease-of-use of the system, and likely with additional training and a more ergonomic prosthesis, she would have been able to accomplish all tasks.

### Device usability and user experience

**Fig. 13:**
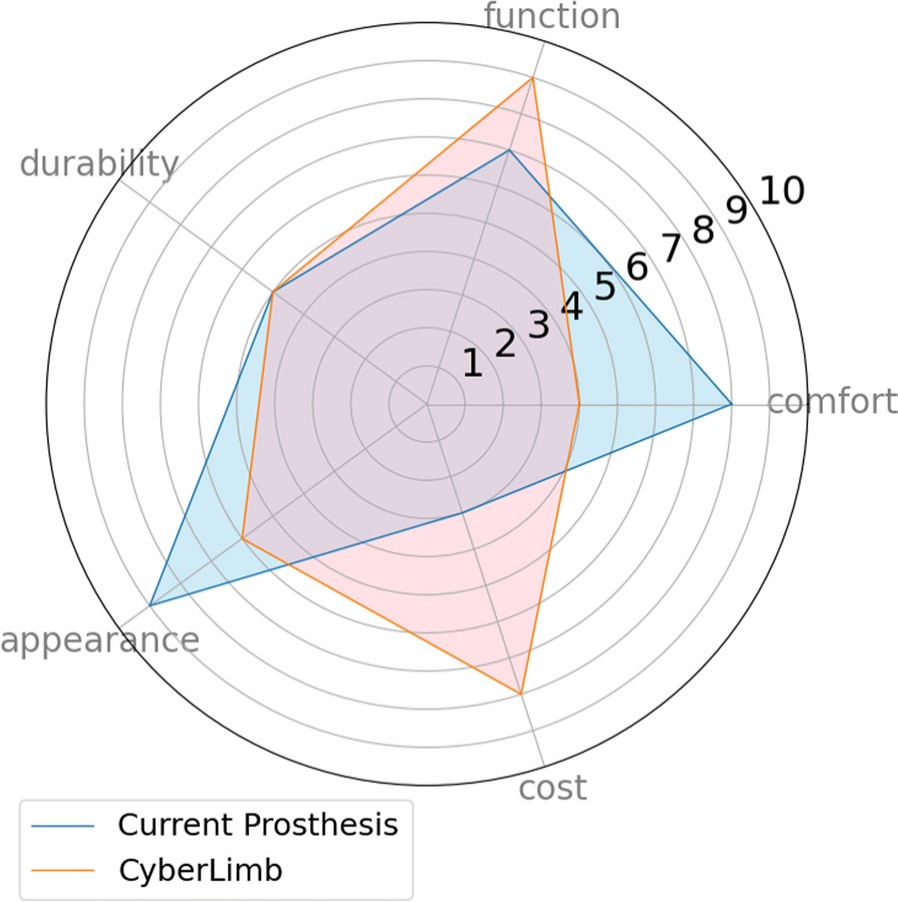
Pilot’s subjective comparison of her current daily prosthesis with the competition prosthesis (0 = “poor”, 5 = “neutral”, 10 = “very good”)

**Fig. 14:**
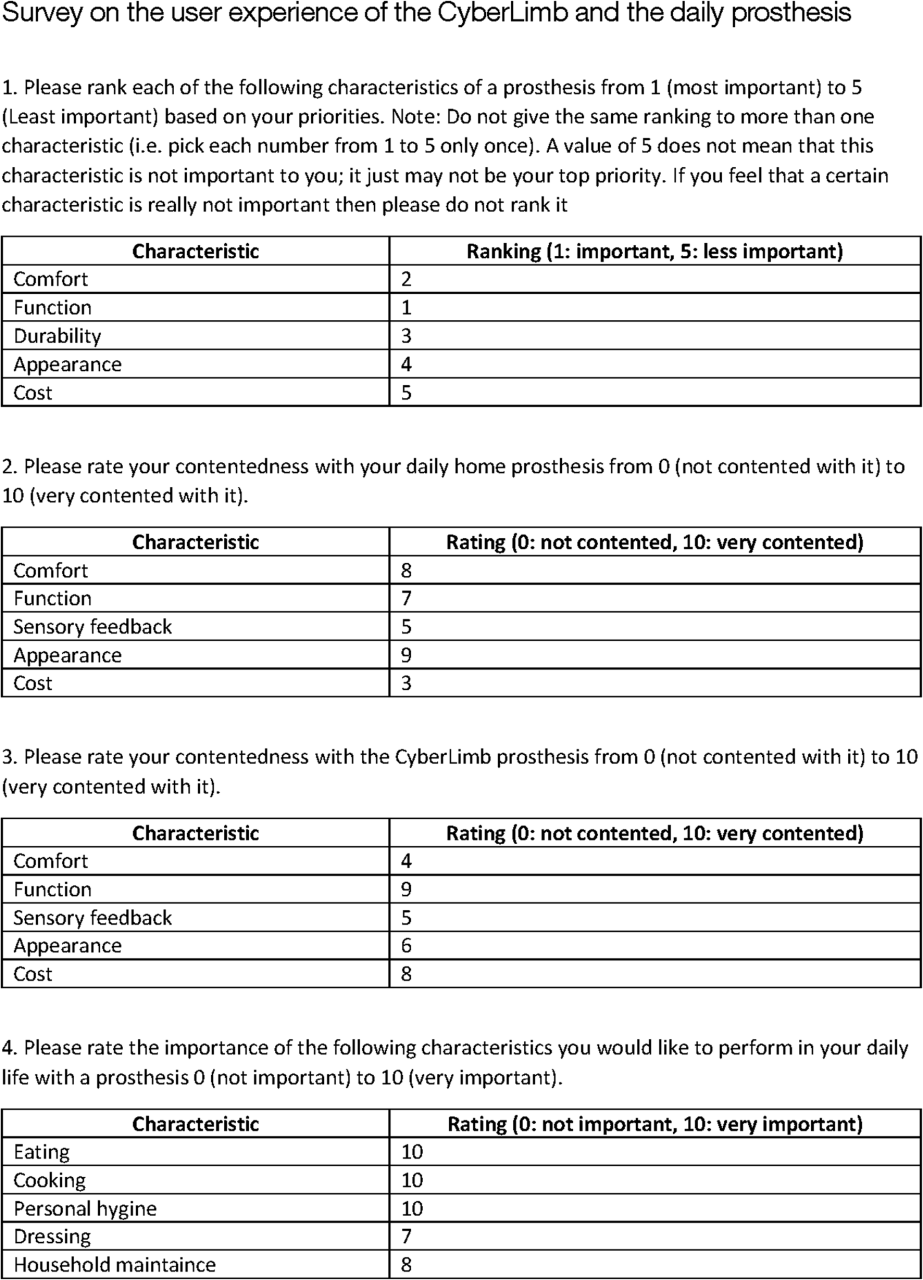
Usability survey on CyberLimb and daily prosthesis page 1 of 2

**Fig. 15:**
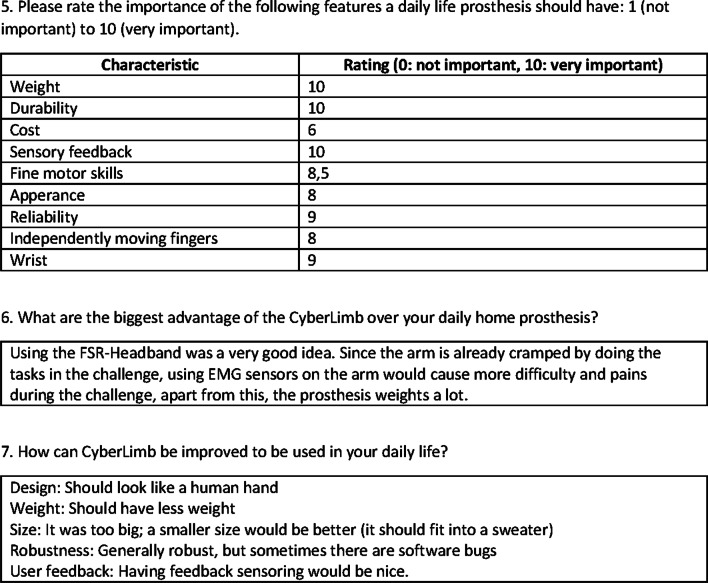
Usability survey on CyberLimb and daily prosthesis page 2 of 2

We developed a novel 3D printed prosthesis, using methods from robotics research and applying them to our prosthesis. To have feedback on device usability and user experience, it was necessary to find common metrics to compare with. In the research of Biddiss et al. [4], 7 studies are analyzed qualitatively. Users of different types of prostheses evaluated the importance of different features. We designed a usability and performance survey with results that were quantified in Fig. 13. The survey (Figs. 14, 15) was inspired by the questionnaire in Biddiss’s thesis [28]. The survey aimed to get user feedback from our pilot on CyberLimb and the pilot’s daily prosthesis and compare it with the results of Biddiss’s research [4]. The survey was conducted via telephone and the questions and answers of our pilot can be found in the appendix (Figs. 14, 15). The results are shown in Tables 2, 3, and 4. Table 2 compares the importance ranking of features of a body-powered and a myoelectric prosthesis with the CyberLimb prosthesis from our pilot’s perspective. The values of the body-powered prosthesis and the myoelectric prosthesis consist of mean values of the Biddiss’s studies based on Friedman’s Rank Test [4]. For our pilot, the functionality and the comfort are the most important features for the Cybathlon challenge. The appearance and the cost, however, are less important for the CP.

Figure 13 shows an assessment of durability, function, comfort, cost and appearances between the pilot’s daily-life prosthesis (DLP) and the CyberLimb competition prosthesis (CP). The pilot felt that the DLP was more comfortable and aesthetically more pleasing. She also felt that CP was less expensive. However, this might have been influenced by the fact that our prosthesis was still a prototype and not market-ready. Since CP featured more functions tailored for the tasks of the Cybathlon challenge, this might have influenced the functionality ranking given to our prosthesis. For this comparison, the DLP was ranked regarding the home tasks and the Cyberlimb was ranked regarding the Cybathlon tasks.

**Table 2:** Ranking of the importance of the design priority (1 = very important, 5 = less important), comparison of the qualitative analysis in the study of Biddiss et al. [4] with the CyberLimb prosthesis

Body-powered prosthesis [4]	Myoelectric [4]	Our pilot’s rating on CyberLimb
Function (2.07)	Comfort (1.91)	Function (1)
Comfort (2.07)	Function (2.39)	Comfort (2)
Durability (3.25)	Appearance (3.01)	Durability (3)
Cost (3.73)	Durability (3.23)	Appearance (4)
Appearance (3.89)	Cost (4.45)	Cost (5)

Similar to the results found in [4], the functionality and the comfort of the prosthesis are the most important factors for our pilot. While the cost is still an important factor, our pilot ranked the appearance higher than the prosthesis’ cost. The results from Table 2 matched our priorities. For a competition prosthesis, the importance of the functionality has to be in the first place. This result shows that the pilot had the same expectations from the prosthesis as the development team of the CyberLimb in the Cybathlon competition context.

A comparison between the pilot’s DLP and the CyberLimb CP based on the five categories is shown in Fig. 13. One significant advantage of the CyberLimb is the functionality, as it offers a gripper control with the FSR headband. This control does not strain the pilot’s arm and allows the pilot to control the prosthesis with less effort. However, it needs to be noted that this actuation mode might have worked well for our pilot exclusively within the competition context and results from our prosthesis weight and features. Another highly ranked feature is the flexibility of the gripper. The gripper is able to grasp all objects from the challenge, from fragile plastic cups to a heavy hammer. The fact that the majority of the CyberLimb device is fully 3D printed, significantly reduces the manufacturing costs compared to existing commercial prostheses. However, the main contributing factor of the price difference is that as a prototype, Cyberlimb does not contain any certification nor clinician evaluation costs that commercial prostheses are required to have in order to be market-ready. Besides, the price of the DLP was not revealed by the pilot, the result is only based on the answer of the pilot. Additionally, CyberLimb was in fact still too heavy for our pilot, and hence its performance rating was relatively low (4/10) within the comfort category. Due to the gripper shape and size of the prosthesis as a whole, the “appearance” category received a 6/10. Generally, our pilot indicated that a prosthesis design similar to an average human arm hand would be preferable. This was one of the current limitations of our proposed device, in which we traded-off appearance with functionality benefits. Since our pilot is able to move and rotate her upper arm, the wrist actuation was good for the competition, however, in order to suffice our pilot’s daily life outside of the Cybathlon challenge, we would need to focus on functionalities specialized on eating, cooking, and personal hygiene, among others, as these are ranked with the highest importance of an arm prosthesis by our pilot.. Lastly, our pilot indicated that having sensory feedback would be beneficial for her everyday life to help her manipulate and control objects. Table 3 depicts common daily tasks that our pilot ranked as important.

**Table 3:** Rating of importance of tasks in which the pilot would like to use the prosthesis in daily life

Task	Rating (from 0 to 10)
Eating	10
Cooking	10
Personal hygiene	10
Dressing	7
Household maintains	8

(0 = “not important”, 10 = “very important”)

Generally, our pilot indicated the strong desire of having a prosthesis, which replaces her lost arm in daily activities as mentioned above. Important requirements for a prospect arm prosthesis specialized for daily life usage, and according to ouor pilot, are shown in Table 4. Functionality and comfort are the most important requirements for designing a prosthesis. The CyberLimb has adaptive gripper fingers and large grasping friction due to the rubber on the gripper surface. The gripper had enough force to grasp all objects. Even though the pilot was able to properly grasp the hammer with the prosthesis and execute the task for a short period of time, the discomfort increased rapidly during that attempt. Therefore, the hammer task exposed that the weight distribution of the prosthesis needed to be shifted more to the upper arm in order to avoid a large leverage effect and that the overall weight needed to be reduced. Ideally, the weight of the whole system would be about 3.3$\%$ of the bodyweight, which is the average weight of a human forearm and hand [29]. Compared to the pilot’s daily prosthesis, weighing 1.03 kg, the CyberLimb had almost twice as much weight at 1.95 kg. Furthermore, the dimensions of our prosthesis were too large causing some issues to our pilot, for instance, the width of the CyberLimb is too large and thus could not fit into the pullover of the laundry task.

**Table 4:** Importance rating of general arm prosthesis properties

Features	Rating (from 0 to 10)
Weight	10
Durability	10
Cost	6
Sensory feedback	10
Fine motor skills	8,5
Appearance	8
Reliability	9
Independently moving fingers	8
Wrist	9

Our pilot indicated that features such as sensory feedback and lightweightness are very important for a prosthesis in daily life (0 = “not important”, 10 = “very important”)

## Discussion

With the CyberLimb prosthesis we developed, we were able to demonstrate the advantages of integrating robotic technology into the design of a prosthesis as a proof-of-concept, e.g. shared control features or 3D printing technology. For instance, it helped us to develop a working and individualized prosthesis in a relatively short period of time (final concept in less than 1 year), as we could base our device on well-established manually actuated gripper-style systems for ease of use. Despite advances in the mechatronic design of prosthesis systems, the control of such systems is still a complex problem with limited results, and thus many prosthesis users still prefer manually actuated systems [30]. Our approach showed that a simple gripper could accomplish the majority of tasks within the Cybathlon competition, and combined with a shared control interface, the prosthesis could be adjusted to solve different tasks. We demonstrated in training sessions that the pilot was able to accomplish every task except the laundry task in the Cybathlon competition, covering a wide range of use cases for the device.

Throughout training sessions with our pilot, we were able to incrementally update the prosthesis design to fit our pilot’s needs and still meet most of the requirements that we initially setup via the storybook analysis. Initially, we incorporated the gripper control via EMG sensors embedded in the stump interface. Based on trained models of our pilot’s attempting to flex and extend our pilot’s wrist, our pilot could control the opening and closing of the gripper. However, due to the limitations of our device, and time constraints, we determined, thorough discussions with our pilot, that a head interface would be a simpler and intuitive solution. Our pilot ultimately preferred the FSR headband solution because it required less physical and cognitive effort to operate. This result was expected as our prosthesis design is much heavier than her current DLP, thereby making it physically difficult to control with EMG. However, with that said, aside from the physical limitations of using EMG with our device, the pilot indicated that cognitively, even with her current DLP, controlling the prosthesis grip can be mentally fatiguing. Flexing her muscles for the decoder to pick up motor intent, which may not necessarily align with her true motor intentions, could be a tiring action for her. Our pilot indicated that the FSR headband was a much simpler solution during the competition. It should be noted, however, that this is based on the feedback from one prosthesis user and could have been influenced by the hardware constraints of our device. Additionally, the FSR headband solution does not necessarily promote an aesthetic appeal for an integrated prosthesis system. However, it provides a foundation for an alternative human-machine interface that is simple to operate. As a possibility, the headband could be modified to be more aesthetically pleasing while still providing the same benefit of control simplicity without the physical and cognitive efforts required to operate current powered prosthesis systems. As an additional option to a human-machine interface based on biological signals, having a method to control the gripper directly (e.g. with a phone interface) could be useful to reduce frustration on pilots in instances when the prosthesis control fails to decode the user’s intentions.

In addition to the FSR headband control interface, we incorporated an automatic wrist control system to share the control with the user. Depending on the selected prosthesis mode from the phone control system, the wrist could automatically adjust the angle based on the prosthesis position in space. The auto-leveling mode allowed the gripper to always be positioned horizontally, regardless of the prosthesis angle. This became particularly useful in cases where the pilot needed to reach up high for an object. We also discovered that in training sessions, she was having difficulty flipping blue cups in the cup stacking task. Normally, our pilot would be able to supinate the prosthesis axially using the other arm, but since it was not possible to touch the prosthesis with our pilot’s other arm to be in compliance with the Cybathlon task rules, we devised an alternative solution together with our pilot to help flip the cups. Based on a relative rotation of the prosthesis after she grasped a cup, the wrist rotated in the opposite direction, thereby flipping the cup for our pilot (using the max auto-leveling mode). Ultimately, this shared control allowed our pilot to successfully complete this task in the competition.

Functionality and comfort of a prosthesis were selected as the most important design priorities by our pilot, irrespective of whether it is a daily life prosthesis or a competition prosthesis. For the Cybathlon competition, where the ultimate goal is finishing all tasks in a short period of time, our pilot was generally satisfied with the functionalities of the CyberLimb (Figs. 14, 15), although the weight and the durability should be improved. Table 4 summarizes the important points from our pilot’s and the author’s perspective for the design of a prosthetic device within the context of the Cybathlon competition and may correlate to the requirements of a prosthetic device for more general daily life tasks. Such points are part of our future work to improve our design.

By working together with the prosthesis user and updating the device based on training session findings, we were able to create an intuitive system to control. Despite providing a simple solution for the prosthesis user, there were several limitations with the current device. However, as previously mentioned, this device serves as a proof-of-concept prototype to establish a relationship with the prosthesis user for further human-centered device development. The prosthesis system proved far too heavy to be used in daily tasks in its current form factor. Additional usability concerns consist of a backpack with cabling connected to the device. Future iterations should minimize the hardware components as much as possible to retain functionality in a lighter and more user-friendly device. Aside from usability concerns, the device was specifically designed for our pilot to accomplish tasks from the Cybathlon competition. While the Cybathlon competition covers a wide range of tasks, this does not necessarily mean that the device will generalize well to all activities of daily living. For instance, the gripper was designed with small notches specifically to grip the scissors from the competition. It is likely there are many other objects that the pilot would have difficulty grasping with only a two-finger gripper design. In the future, we will need to consider similar soft gripper mechanisms that could incorporate additional grips, such as a lateral key pinch. Furthermore, the solution we developed for the task of the haptic box cannot be transferred to everyday life situations as well. Rather, it can be seen as a functional proof-of-concept for incorporating machine intelligence to solve a specific and clearly defined task. The results on the test data set promised a high potential of this approach for the competition. However, a resulting sensor failure before the competition showed the low robustness of this approach. Despite some of these limitations, we were successful in establishing a user-centric control system based on a simple gripper-style prosthesis.

## Conclusion

Together with our pilot, we developed a practical and intuitive prosthesis system to be used in daily tasks resembling those of the Cybathlon competition. The simple gripper design minimizes the need for complex control measures allowing the user to accomplish tasks with ease and consistency. The FSR headband interface allowed our pilot to retain full control of the arm without the need for focusing on complex muscle activation patterns. An automatic wrist flexion and extension control shared the task load with the user to provide an intuitive gripper angle for grasping objects. The manual forearm supination and pronation provided an additional degree of flexibility to adapt the gripper position when needed without requiring a full-body rotation. Overall, the system provided a proof-of-principle prototype of a simple-to-use yet highly versatile prosthetic device.

Preparing for the Cybathlon competition challenged our team to develop a task-oriented system while focusing on meeting user needs. We aimed at keeping our pilot in the prosthesis development loop to make better decisions and create novel control methods that were easy to operate during the competition. In future competitions, we will focus on refining our device design to be more robust and generalizable, while still providing the intuitive control interface for the prosthesis user. Ultimately, the outcome of the competition for our team was a prosthetic concept, which is simple to use and was able to complete most of the Cybathlon competition tasks. We believe insights of our concept have the possibility to expand prosthetics research into new directions, and contribute to an overall improvement of prosthetic devices in general.

## Data Availability

The datasets used and/or analysed during the current study are available from the corresponding author on reasonable request.

## Abbreviations

BLDC: Brushless direct current
CP: Competition prosthesis
DLP: Daily-life prosthesis
DoA: Degrees of actuation
DoF: Degrees of freedom
EMG: Electromyography
HD: Harmonic drive
HDMI: High definition multimedia
IMU: Inertial measurement unit
MLP: Multilayer perceptron
mNm: Millinewtonmeters
PCB: Printed Circuit Board
rpm: Round per minute
sEMG: Surface electromyography
TPU: Thermoplastic polyurethane
UI: User interface
